# High quality draft genome sequence of the moderately halophilic bacterium *Pontibacillus yanchengensis* Y32^T^ and comparison among *Pontibacillus* genomes

**DOI:** 10.1186/s40793-015-0085-y

**Published:** 2015-11-10

**Authors:** Jing Huang, Zi xu Qiao, Jing wei Tang, Gejiao Wang

**Affiliations:** State Key Laboratory of Agricultural Microbiology, College of Life Sciences and Technology, Huazhong Agricultural University, Wuhan, 430070 P. R. China

**Keywords:** *Pontibacillus*, *Pontibacillus yanchengensis*, Genomic comparison, Moderately halophilic, Flagellar and chemotaxis

## Abstract

*Pontibacillus yanchengensis* Y32^T^ is an aerobic, motile, Gram-positive, endospore-forming, and moderately halophilic bacterium isolated from a salt field. In this study, we describe the features of *P. yanchengensis* strain Y32^T^ together with a comparison with other four *Pontibacillus* genomes. The 4,281,464 bp high-quality-draft genome of strain Y32^T^ is arranged into 153 contigs containing 3,965 protein-coding genes and 77 RNA encoding genes. The genome of strain Y32^T^ possesses many genes related to its halophilic character, flagellar assembly and chemotaxis to support its survival in a salt-rich environment.

**Electronic supplementary material:**

The online version of this article (doi:10.1186/s40793-015-0085-y) contains supplementary material, which is available to authorized users.

## Introduction

*Pontibacillus yanchengensis* Y32^T^ (= CGMCC 1.10680^T^ = CCTCC AB209311^T^ = NRRL B-59408^T^) was isolated from a salt field in Yancheng, China [1], and affiliated to the family *Bacillaceae*, order *Bacillales*, phylum *Firmicutes* [2, 3]. The genus *Pontibacillus* means “*Bacillus* pertaining to the sea” and was first identified by Lim *et al.* in 2005 [4]. To date, the genus contains six species, including *Pontibacillus yanchengensis* [1], *Pontibacillus chungwhensis* [4], *Pontibacillus marinus* [5], *Pontibacillus halophilus* [6], *Pontibacillus litoralis* [7], and *Pontibacillus salicampi* [8], which are isolated from a salt field, a solar saltern, a solar saltern, a sea urchin, a sea anemone, and a saltern soil, respectively.

The *Pontibacillus* members are characterized as moderately halophilic, Gram-positive, aerobic, endospore-forming and rod-shaped bacteria. They are motile by peritrichous flagella and their DNA has a low G + C content. They are able to survive in salt-rich environments and grow optimally at 5-20 % NaCl (w/v) [9]. To adapt to saline environments, halophilic microorganisms have developed various biochemical strategies to maintain cell function, such as induction of Na^+^/H^+^ antiporter systems and the production of compatible solutes. The compatible solutes are gaining increasing interest since they can be used as stabilizers, salt antagonists, or stress-protective agents [10–13]. In addition, a *Pontibacillus* strain could produce biosurfacants which is useful in degradation of paraffinic mixture or saline organic contamination [11].

In this study, we sequenced five *Pontibacillus* type strains, including *P. yanchengensis* Y32^T^*,**P. chungwhensis* BH030062^T^, *P. marinus* BH030004^T^, *P. halophilus* JSM076056^T^ and *P. litoralis* JSM072002^T^(The GenBank accession summary of the strains is shown in Additional file 2). Here we present the draft genome sequence of *P. yanchengensis* Y32^T^ and compare it to the genomes of four other type strains. To the best of our knowledge, this is the first description of the *Pontibacillus* genome.

## Organism information

### Classification and features

**Fig. 1:**
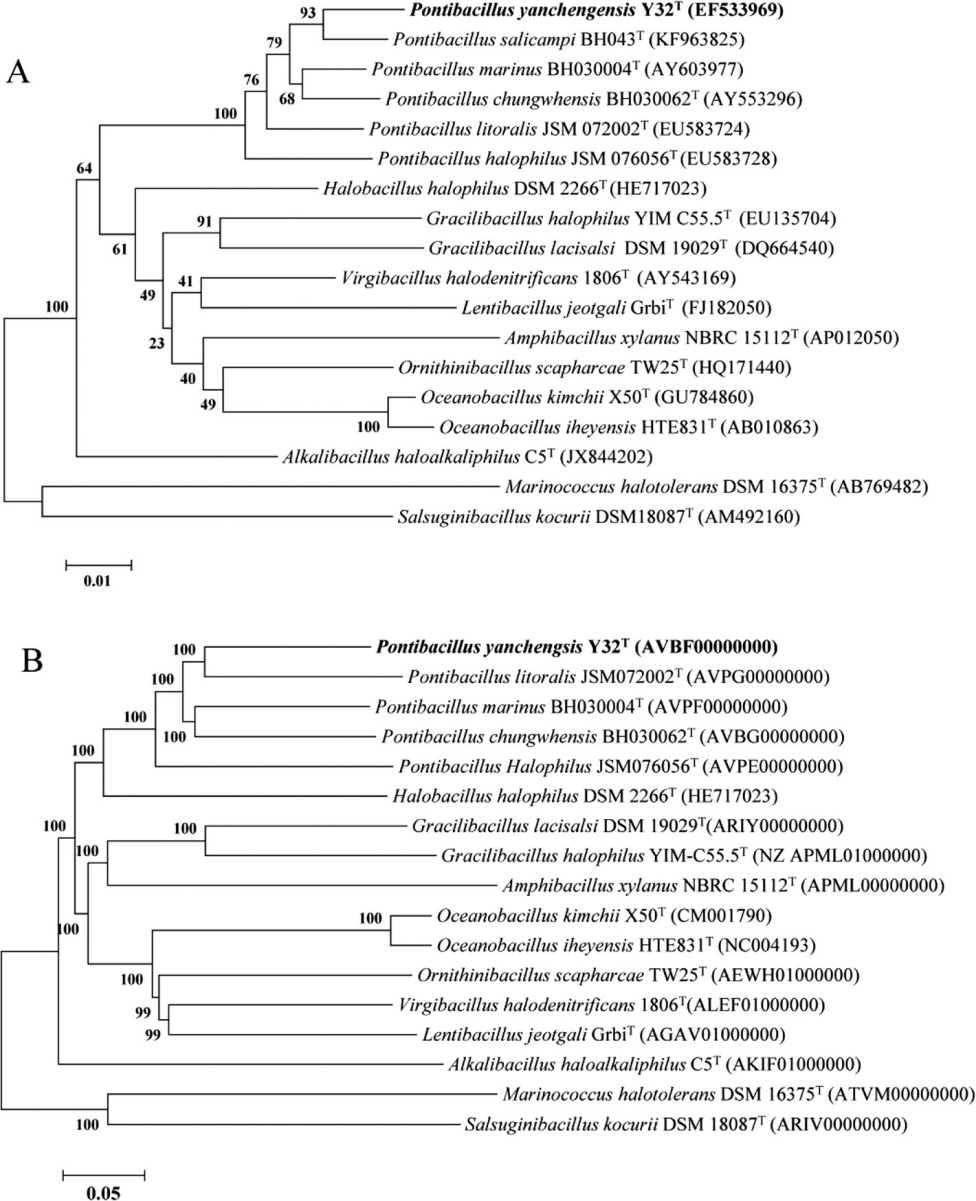
Phylogenetic analysis. **a** The 16S rRNA gene-based phylogenetic tree showing the position of *P. yanchengensis* Y32^T^. **b** The NJ phylogenetic tree of *P. yanchengensis* Y32^T^ relative to 16 genome-sequenced strains from the *Bacillaceae* family was built based on the core protein sequences. All genome FASTA files were downloaded from NCBI except for the *Pontibacillus* genus. A total of 602 conserved proteins were identified using the cluster algorithm tool OrthoMCL [16, 17]. The phylogenetic trees were constructed using the neighbor-joining method by MEGA 6.0 software [15] with a bootstrap value of 1,000

*P. yanchengensis* Y32^T^ was isolated from a salt field in Yancheng prefecture, on the east Yellow Sea in China. A taxonomic analysis was conducted based on the 16S rRNA gene sequence. The representative 16S rRNA gene sequences of the most closely related strains were downloaded from NCBI and multi-aligned by CLUSTAL W [14]. Phylogenetic consensus trees were constructed based on the aligned gene sequences using the neighbor-joining method with 1,000 bootstraps by using MEGA 6.0 [15]. The phylogenetic tree based on the 16S rRNA gene sequences indicated that strain Y32^T^ was clustered within a branch containing other species in the genus *Pontibacillus* (Fig. 1a).

Seventeen related strains of *Bacillaceae* [2] with complete genome sequences were chosen for further phylogenetic analysis, including the four draft-genome sequences of *Pontibacillus* that were sequenced by us. In total, 602 core protein sequences were extracted using the cluster algorithm tool OrthoMCL [16, 17] with default parameters. The neighbor-joining (NJ) phylogenetic tree showed that the five *Pontibacillus* species clustered into the same branch (Fig. 1b), which was in accordance with the 16S rRNA gene-based phylogeny (Fig. 1a).

**Fig. 2:**
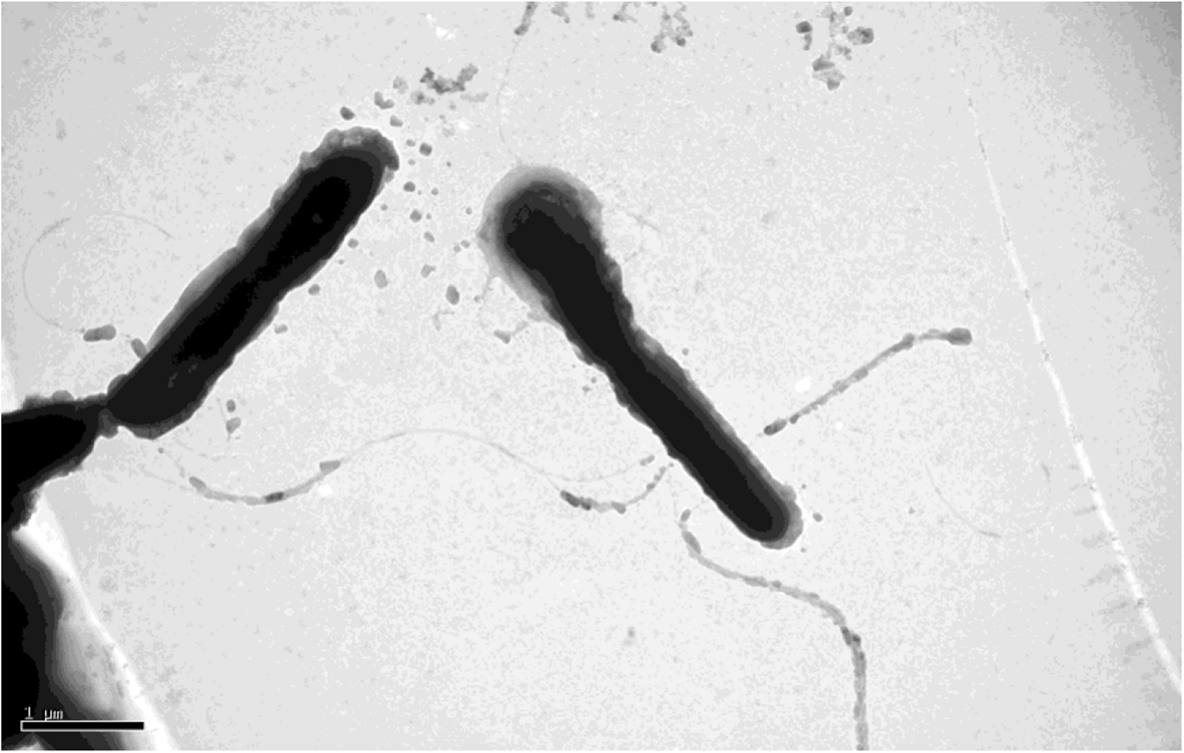
A transmission micrograph of *P. yanchengensis* Y32^T^. The scale bar represents 1 μm. Strain Y32^T^ was cultured aerobically on Bacto marine broth 2216 (Difco) agar plus 5 % NaCl at 37C° for 48 h

*P. yanchengensis* Y32^T^ is Gram-positive, rod-shaped (0.5–0.9 × 1.9–2.5 μm), motile with flagella (Fig. 2) and endospore-forming. It can grow on Bacto marine broth 2216 (Difco) agar medium containing 3–20 % (w/v) NaCl and does not grow in the absence of NaCl [1]. The optimal growth temperature for Y32^T^ is 35–40 °C (Table 1). The strain is oxidase- and catalase-positive and negative for the production of H_2_S or indole. It has been reported to reduce nitrate to nitrite [1]. *P. yanchengensis* Y32^T^ can use a few kinds of sole carbon sources, including D-glucose, D-fructose, D-mannitol, D-maltose and D-trehalose [1]. Compared to the other *Pontibacillus* genus type strains, only *P. yanchengensis* Y32^T^ can utilize D-mannitol as sole carbon source [1]. KEGG pathway analysis of the five *Pontibacillus* genomes (see below) revealed that only strain Y32^T^ had the key enzyme mannitol-1-phosphate 5-dehydrogenase (gene ID: N782_14920) which could potentially catalyze D-mannitol 1-phosphate to D-fructose 6-phosphate. This result was consistent with the phenotype. As one of the most abundant polyols in nature, mannitol metabolism provides an important physiologic contribution in microbial stress responses [18].

**Table 1:** Classification and general features of *P. yanchengensis* Y32^T^ according to the MIGS recommendations [26]

MIGS ID	Property	Term	Evidence code
	Current classification	Domain *Bacteria*	TAS [27]
		Phylum *Firmicutes*	TAS [28]
		Class *Bacilli*	TAS [29, 30]
		Order *Bacillales*	TAS [2, 3]
		Family *Bacillaceae*	TAS [2, 3]
		Genus *Pontibacillus*	TAS [4]
		Species *Pontibacillus yanchengensis*	TAS [1]
		Type strain Y32^T^	TAS [1]
	Gram stain	Positive	TAS [1]
	Cell shape	Rod-shaped	TAS [1]
	Motility	Motile	TAS [1]
	Sporulation	Sporulating	TAS [1]
	Temperature range	15–45 °C	TAS [1]
	Optimum temperature	35–40 °C	TAS [1]
	Salinity	3–20 % (w/v)	TAS [1]
	Optimum salinity	6–8 % (w/v)	TAS [1]
	pH range	6–9.5	TAS [1]
	Optimum pH	7–8	TAS [1]
MIGS-22	Oxygen	aerobic	TAS [1]
MIGS-15	Biotic relationship	Free-living	NAS
MIGS-6	Habitat	Salt-field	TAS [1]
MIGS-14	Pathogenicity	Non-pathogenic	NAS
MIGS-4	Geographic location	Yancheng, China	TAS [1]
MIGS-4.1	Latitude	N32°23'	TAS [1]
MIGS-4.2	Longitude	E120°0'	TAS [1]
MIGS-4.3	Depth	5–15 cm	TAS [1]
MIGS-4.4	Altitude	Not reported	
MIGS-5	Sample collection time	2011	TAS [1]

Evidence codes - TAS: Traceable Author Statement (i.e., a direct report exists in the literature); NAS: Non-traceable Author Statement (i.e., not directly observed for the living, isolated sample, but based on a generally accepted property for the species, or anecdotal evidence). These evidence codes are from the Gene Ontology project [31]

#### Chemotaxonomic data

When grown on Bacto marine broth 2216 (Difco) agar medium plus 3 % (w/v) NaCl, *P. yanchengensis* Y32^T^ contained anteiso-C_15:0_, iso-C_15:0_, and iso-C_14:0_ as the major fatty acids and menaquinone (MK-7) as the predominant respiratory quinone. The cell wall peptidoglycan type was *meso*-diaminopimelic [1]. The classification and general features of *P. yanchengensis* Y32^T^ are shown in Table 1.

## Genome sequencing information

### Genome project history

*P. yanchengensis* Y32^T^ was selected for sequencing on the basis of its taxonomic representativeness, halophilic features and potential industrial applications. Genome sequencing was performed by Majorbio Bio-pharm Technology Co., Ltd., Shanghai, China. The draft genome sequence was deposited in NCBI with contigs larger than 200 bp. The GenBank accession number is AVBF00000000. A summary of the genome sequencing project information is shown in Table 2.

**Table 2:** Genome sequencing project information for *P. yanchengensis* Y32^T^

MIGS ID	Property	Term
MIGS-31	Finishing quality	High-quality draft
MIGS-29	Libraries used	Illumina Paired-End library (300 bp insert size)
MIGS-29	Sequencing platform	Illumina Hiseq2000
MIGS-31.2	Sequencing coverage	186.5 x
MIGS-30	Assemblers	SOAP denovo v1.05
	Gene calling method	GeneMarkS+
	GenBank date of release	November 6, 2014
	GenBank ID	AVBF00000000
	Locus Tag	N782
MIGS-32	BIOPROJECT	PRJNA214569
	BioSample	SAMN02650962
MIGS-13	Source material identifier	Y32^T^
	Project relevance	Genome comparison

### Growth conditions and DNA isolation

*P. yanchengensis* Y32^T^ was grown aerobically in 50 mL Bacto marine broth 2216 (Difco) plus 5 % NaCl (w/v) at 37 °C for 2 d with 150 rpm shaking. Cells were harvested by centrifugation and a pellet with an approximate wet weight of 20 mg was obtained. The genomic DNA was extracted using the QIAamp DNA kit according to the manufacturer’s instructions (Qiagen, Germany). The quality and quantity of total DNA was determined using a NanoDrop Spectrophotometer 2000. Five micrograms of genomic DNA was sent to Majorbio (Shanghai, China) for sequencing on a Hiseq2000 (Illumina, CA) sequencer.

### Genome sequencing and assembly

The Illumina Hiseq2000 technology of Paired-End (PE) library with an average insert size of 300 bp was used to determine the sequence of *P. yanchengensis* Y32^T^. A total of 4,083,912 × 2 high quality reads totaling 824,950,224 bp of data with an average coverage of 186.5 x was generated. Raw reads were filtered using a FastQC toolkit followed by assembly with SOAP denovo v1.05 and optimizing through local gap filling and base correction with Gap Closer.

### Genome annotation

The draft genome sequence was deposited at NCBI and was annotated through the Prokaryotic Genome Annotation Pipeline, which combined the Best-Placed reference protein set and the gene caller GeneMarkS+. The WebMGA server was used to identify the Clusters of Ortholog Groups [19]. Transmembrane helices and signal peptides were predicted by the online bioinformatic tools TMHMM 2.0 [20, 21] and SignalP 4.1 [22], respectively.

## Genome properties

The final whole genome of *P. yanchengensis* Y32^T^ was 4,283,464 bp long, distributed in 153 contigs, and had an average GC content of 39.11 %. Of the total 4,080 predicted genes, 3,965 were protein-coding genes (CDSs), and 77 were RNA genes. A total of 2,615 CDSs (65.95 %) were assigned putative functions, and the remaining proteins were annotated as hypothetical proteins. The genome properties and statistics are summarized in Table 3. The distribution of genes into COGs functional categories is shown in Table 4.

**Table 3:** Genome statistics for *P. yanchengensis* Y32^T^

Attribute	Value	% of Total^a^
Genome size (bp)	4,281,464	100.00
DNA coding region (bp)	3.472,267	81.10
DNA G + C content (bp)	1,674,480	39.11
Number of contigs	153	-
Contig N50 (bp)	55350	-
Total genes	4080	100.00
Protein-coding genes	3965	97.18
RNA genes	77	1.89
Pseudo genes	38	0.93
Frame shifted genes	12	-
Genes with function prediction	2615	65.95
Genes assigned to COGs	2972	74.95
Genes with Pfam domains	3135	79.07
Genes with signal peptides	242	6.10
Genes with transmembrane helices	1176	29.66
CRISPR repeats	0	-

^a^The total is based on either the size of the genome in base pairs or the total number of protein coding genes in the annotated genome

**Table 4:** Number of protein-coding genes associated with the 25 general COG functional categories in the *P. yanchengensis* Y32^T^ genome

COG class	Count	% age^a^	COG description
J	171	4.31	Translation, ribosomal structure and biogenesis
A	0	0.00	RNA processing and modification
K	258	6.51	Transcription
L	148	3.73	Replication, recombination and repair
B	2	0.05	Chromatin structure and dynamics
D	36	0.91	Cell cycle control, cell division, chromosome partitioning
Y	0	0.00	Nuclear structure
V	54	1.36	Defense mechanisms
T	222	5.60	Signal transduction mechanisms
M	173	4.36	Cell wall/membrane/envelope biogenesis
N	71	1.79	Cell motility
Z	0	0.00	Cytoskeleton
W	0	0.00	Extracellular structures
U	58	1.46	Intracellular trafficking, secretion, and vesicular transport
O	119	3.00	Posttranslational modification, protein turnover, chaperones
C	199	5.02	Energy production and conversion
G	253	6.38	Carbohydrate transport and metabolism
E	295	7.44	Amino acid transport and metabolism
F	94	2.37	Nucleotide transport and metabolism
H	132	3.33	Coenzyme transport and metabolism
I	129	3.25	Lipid transport and metabolism
P	186	4.69	Inorganic ion transport and metabolism
Q	71	1.79	Secondary metabolites biosynthesis, transport and catabolism
R	433	10.92	General function prediction only
S	353	8.9	Function unknown
-	508	12.81	Not in COGs

^a^The percentage is based on the total number of protein-coding genes in the annotated genome

### Insights from the genome sequence

**Fig. 3:**
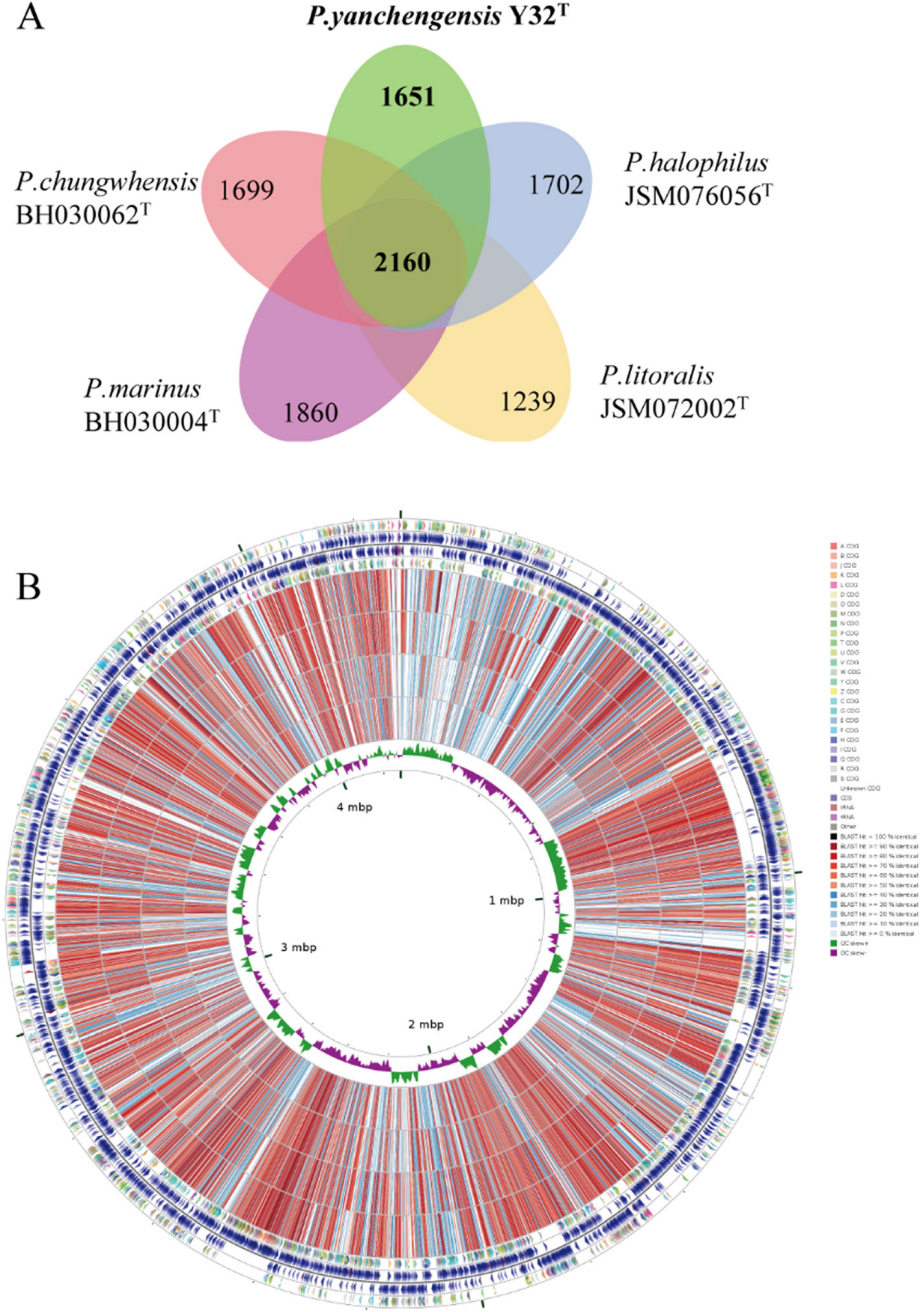
Comparative genomic analysis of the genus *Pontibacillus*. **a** The flower plot shows the numbers of species-specific genes found in each genome of each species (in the petals) and the core orthologous gene number (in the center) of *Pontibacillus*. **b** Comparison map of strain *P. yanchengensis* Y32^T^ and the other four sequenced *Pontibacillus* strains. From outside to inside: rings 1, 4 show protein-coding genes colored by COG categories on the forward/reverse strand, respectively; rings 2, 3 represent genes on the forward/reverse strand, respectively; rings 5, 6, 7, 8 denote the CDS vs CDS BLAST results of *P. marinus* BH030004^T^, *P. chungwhensis* BH030062^T^, *P. halophilus* JSM076056^T^, and *P. litoralis* JSM072002^T^, respectively; ring 9 shows the GC skew

In this study, we compared the genome sequence of *P. yanchengensis* Y32^T^ with the genomes of *P. chungwhensis* BH030062^T^, *P. halophilus* JSM076056^T^, *P. marinus* BH030004^T^ and *P. litoralis* JSM072002^T^. The general features of the five genomic sequences are summarized in Table 5. The results of the core genome analysis suggested that the five *Pontibacillus* species share 2,160 core genes, and *P. yanchengensis* Y32^T^ possesses 1,651 unique genes (Fig. 3a). Among the 1,651 unique genes for strain Y32^T^, 1,154 unique genes were classified into 20 COG functional categories, which mainly belonged to the general function prediction group, the carbohydrate transport, the metabolism group and the function unknown group. The remaining 590 unique genes were not classified into any COG categories (Additional file 1: Table S1). The CG View Comparison Tool [23] was used to draw a comparison graphical circular map of the five *Pontibacillus* strains (Fig. 3b).

**Table 5:** General features of the five *Pontibacillus* genome sequences

Organism	Source	Genome size (bp)	G + C%	Contigs	Contigs N50 (bp)	Genes	CDS	RNA	GenBank No.	CRISPR
*P. yanchengensis* Y32^T^	Salt field	4,283,159	39.11	153	55,350	4,080	3,965	77	AVBF00000000	0
*P. chungwhensis* BH030062^T^	Solar saltern	3,873,758	40.76	40	225,560	3,801	3,685	62	AVBG00000000	0
*P. marinus* BH030004^T^	Solar saltern	4,275,582	38.48	186	45,534	4,329	4,253	52	AVPF00000000	0
*P. halophilus* JSM076056^T^	Sea urchin	3,694,752	42.85	68	190,345	3,653	3,560	58	AVPE00000000	0
*P. litoralis* JSM072002^T^	Sea anemone	3,205,664	38.18	97	112,260	3,282	3,202	49	AVPG00000000	3

All the *Pontibacillus* species were isolated from salty environments. They were characterized as moderately halophilic and cannot grow in the absence of NaCl. As moderate halophiles, effective establishment of ionic and osmotic equilibrium was important for survival in a saline environment. The genome comparison analysis showed that the five *Pontibacillus* strains possessed genes encoding cation/proton antiporter (e.g., Na^+^/H^+^ antiporter, Na^+^/Ca^2+^ antiporter), which played a role in tolerance to high concentrations of Na^+^, K^+^, Li^+^ and/or alkali (Additional file 1: Table S2). Numerous studies showed that Na^+^/H^+^ antiporters play important roles in the pH and Na^+^ homeostasis of cells [24, 25]. Meanwhile, the prediction of the membrane helices of the *P. yanchengensis* Y32^T^ genome suggested that nearly 30% of the genes had transmembrane helix structures (Table 3), which may be involved in ion transport.

**Fig. 4:**
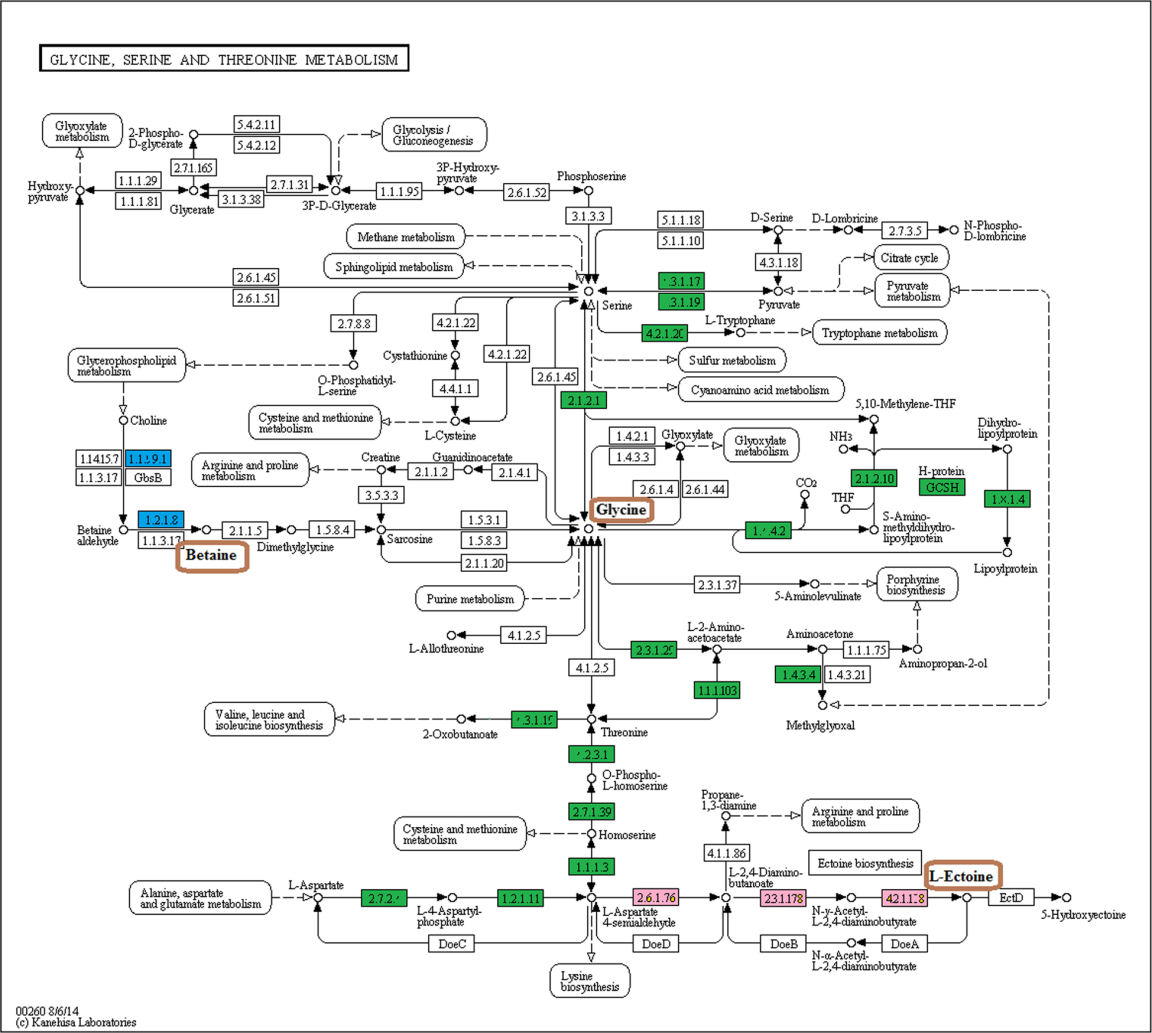
The glycine, serine and threonine metabolic pathways of the five *Pontibacillus* strains (including *P. yanchengensis* Y32^T^, *P. marinus* BH030004^T^, *P. chungwhensis* BH030062^T^, *P. halophilus* JSM076056^T^, and *P. litoralis* JSM072002^T^) reconstructed by KEGG. The green box represents the enzyme shared by all five strains to synthesize glycine. The blue boxes denote the enzymes involved in betain synthesis, which were found in *P. yanchengensis* Y32^T^, *P. chungwhensis* BH030062^T^ and *P. marinus* BH030004^T^. The pathway with pink boxes is found only by *P. marinus* BH030004^T^ and is related to ectoine synthesis

Other than ion transport, the synthesis of compatible solutes (e.g., betaine, ectoine, amino acids) was beneficial for survival under extreme osmotic stress. Many compatible solute synthesis-related genes were identified in the genomes of the five *Pontibacillus* species (Additional file 1: Table S2). The Kyoto Encyclopedia of Genes and Genomes was used to reconstruct the glycine, serine and threonine metabolic pathways (Fig. 4). The metabolic pathways suggested that the five *Pontibacillus* strains could synthesize glycine as the main compatible solute. In addition, *P. yanchengensis* Y32^T^, *P. chungwhensis* BH030062^T^ and *P. marinus* BH030004^T^ could synthesize betaine through the precursor choline. *P. marinus* BH030004^T^ also possessed the pathway of ectoine synthesis. These results indicated that the five *Pontibacillus* species use different strategies to cope with osmotic stress.

**Fig. 5:**
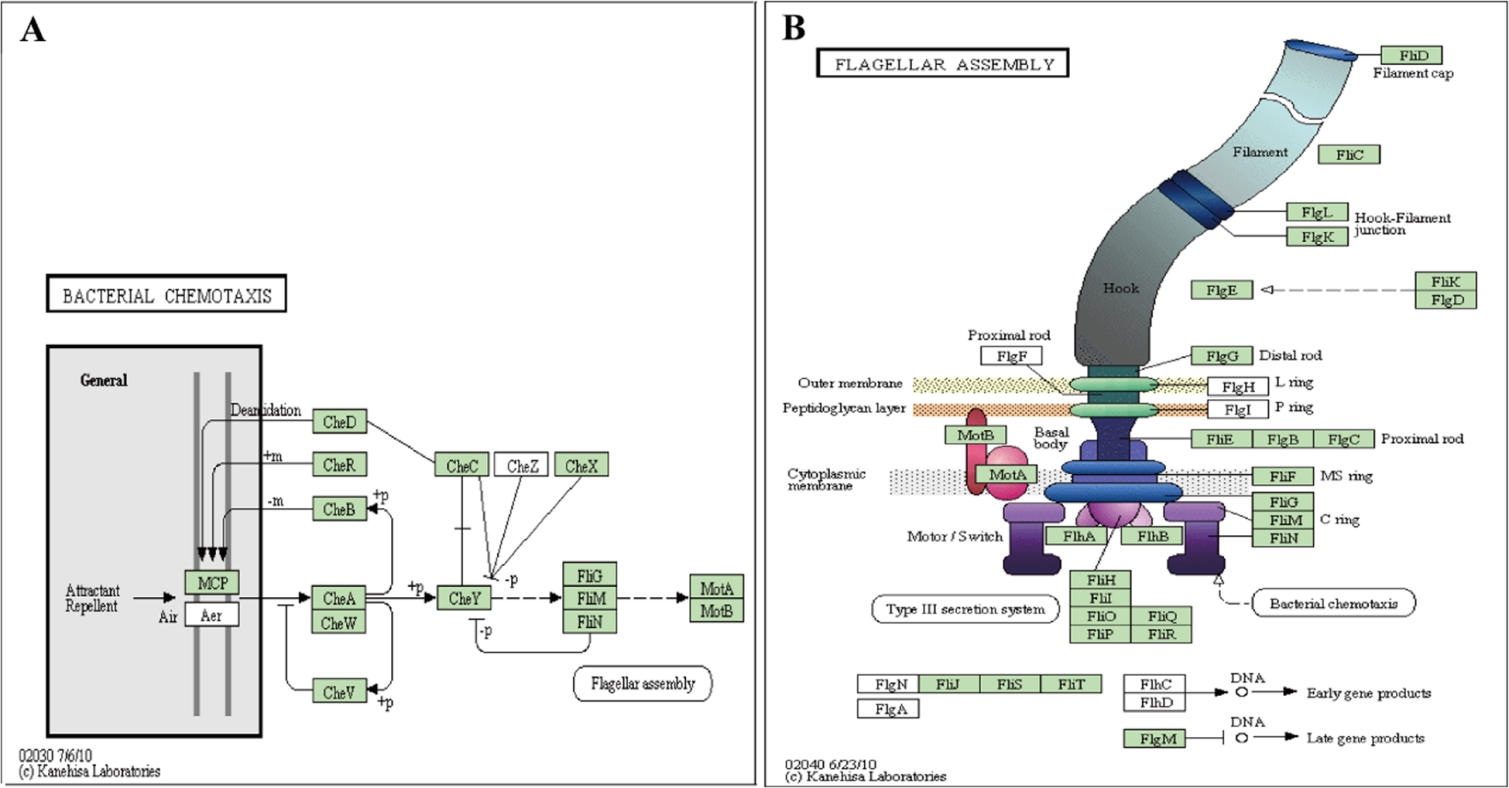
Reconstructed bacterial chemotaxis (**a**) and flagellar assembly (**b**) KEGG map of the five *Pontibacillus* strains. Green boxes represent the chemotaxis- and flagellar-related protein-coding genes identified in all five *Pontibacillus* genomes (*P. yanchengensis* Y32^T^, *P. marinus* BH030004^T^, *P. chungwhensis* BH030062^T^, *P. halophilus* JSM076056^T^, and *P. litoralis* JSM072002^T^)

Many flagella-related genes were identified in the genomes of the five *Pontibacillus* species. Reconstruction of a multi-organism KEGG map suggested that the five *Pontibacillus* strains had intact chemotaxis systems (Fig. 5a) and flagella assembly-related genes (*flg*, *fli* and *flh*) (Fig. 5b). The moderately halophilic *Pontibacillus* strains were unable to grow with NaCl as the sole salt unless artificial seawater was added [1, 4–8]. Flagella and chemotaxis may play important roles in response to environmental salts.

## Conclusions

This study provided genomic information for *P. yanchengensis* strain Y32^T^ and the comparison of five *Pontibacillus* genomes. Strain Y32^T^ has functional genes encoding cation/proton antiporters and proteins for biosynthesis of compatible solutes such as glycine and ectoine. Compatible solutes could be of use in the cosmetic and food industries [13]. The comparative genomic analysis suggested that the five *Pontibacillus* strains possess different synthetic pathways for compatible solutes which provided diverse applications of the strains.

## Additional files

Additional file 1: Table S1. COG functional categories of the 1651 genes unique to *P.yanchengensis* Y32^T^. **Table S2** Species distribution analysis of osmotic stress related gene families. (DOCX 15 kb) Additional file 2:
**GenBank Accession Summary, Strain ID Summary, Reference Search Summary.** (DOC 33 kb)
