# Supplementary material for: High quality draft genome sequence of the moderately halophilic bacterium Pontibacillus yanchengensis Y32T and comparison among Pontibacillus genomes
Source: Stand Genomic Sci. 2015 Nov 10;10:93. doi: 10.1186/s40793-015-0085-y (PMC4641356; doi:10.1186/s40793-015-0085-y)
Supplement: Additional file 1: Table S1. — COG functional categories of the 1651 genes unique to P.yanchengensis Y32T. Table S2 Species distribution analysis of osmotic stress related gene families. (DOCX 15 kb) [file 40793_2015_85_MOESM1_ESM.docx]

Additional file 1

**Table S1** COG functional categories of the 1651 genes unique to *P.yanchengensis* Y32^T^

| COG class | Count | %age^a^ | COG description |
| --- | --- | --- | --- |
| C | 38 | 3.58 | Energy production and conversion |
| D | 3 | 0.28 | Cell cycle control, cell division, chromosome partitioning |
| E | 66 | 6.22 | Amino acid transport and metabolism |
| F | 23 | 2.17 | Nucleotide transport and metabolism |
| G | 130 | 12.25 | Carbohydrate transport and metabolism |
| H | 30 | 2.83 | Coenzyme transport and metabolism |
| I | 38 | 3.58 | Lipid transport and metabolism |
| J | 22 | 2.07 | Translation, ribosomal structure and biogenesis |
| K | 94 | 8.86 | Transcription |
| L | 39 | 3.68 | Replication, recombination and repair |
| M | 57 | 5.37 | Cell wall/membrane/envelope biogenesis |
| N | 20 | 1.89 | Cell motility |
| O | 25 | 2.36 | Posttranslational modification, protein turnover, chaperones |
| P | 49 | 4.62 | Inorganic ion transport and metabolism |
| Q | 30 | 2.83 | Secondary metabolites biosynthesis, transport and catabolism |
| R | 156 | 14.7 | General function prediction only |
| S | 121 | 11.4 | Function unknown |
| T | 80 | 7.54 | Signal transduction mechanisms |
| U | 12 | 1.13 | Intracellular trafficking, secretion, and vesicular transport |
| V | 28 | 2.64 | Defense mechanisms |
|  | 590 | 35.7 | Not in COGs |
| In total | 1651 |  | |

a The percentage is based on the total number of unique gene of strain Y32^T^.

**Table S2** Species distribution analysis of osmotic stress related gene families

| Gene subsystem | Number of genes | | | | |
| --- | --- | --- | --- | --- | --- |
|  | *P. yanchengsis* Y32^T^ | *P. chungwhensis* BH030062^T^ | *P. halophilus* JSM076056^T^ | *P. litoralis* JSM072002^T^ | *P. marinus* BH030004^T^ |
| Sodium:proton antiporter | 19 | 20 | 17 | 17 | 18 |
| Osmoregulation | 0 | 1 | 1 | 1 | 0 |
| Choline and betaine uptake and betaine biosynthesis | 21 | 23 | 13 | 10 | 34 |
| Ectoine biosynthesis and regulation | 1 | 0 | 0 | 0 | 3 |
| Synthesis of osmoregulated periplasmic glucans | 0 | 0 | 0 | 0 | 1 |
