# Supplementary material for: High quality draft genome sequence of the moderately halophilic bacterium Pontibacillus yanchengensis Y32T and comparison among Pontibacillus genomes
Source: Stand Genomic Sci. 2015 Nov 10;10:93. doi: 10.1186/s40793-015-0085-y (PMC4641356; doi:10.1186/s40793-015-0085-y)
Supplement: Additional file 2: — GenBank Accession Summary, Strain ID Summary, Reference Search Summary. (DOC 33 kb) [file 40793_2015_85_MOESM2_ESM.doc]

**GenBank Accession Summary**

| GenBank Accession | Summary |
| --- | --- |
| AVPG00000000 | AVPG00000000.1 is a bacterial sequences record containing linear, double-stranded DNA (97 bases) from Pontibacillus litoralis JSM 072002 strain JSM 072002. The record was created on November 12, 2014. It contains 1 feature. |
| AVPE00000000 | AVPE00000000.1 is a bacterial sequences record containing linear, double-stranded DNA (68 bases) from Pontibacillus halophilus JSM 076056 = DSM 19796 strain JSM 076056. The record was created on November 12, 2014. It contains 1 feature. |
| PRJNA214569 | BioProject PRJNA214569: http:z//www.ncbi.nlm.nih.gov/bioproject/PRJNA214569 |
| AVBG00000000 | AVBG00000000.1 is a bacterial sequences record containing linear, double-stranded DNA (40 bases) from Pontibacillus chungwhensis strain BH030062. The record was created on November 3, 2014. It contains 1 feature. |
| AVPF00000000 | AVPF00000000.1 is a bacterial sequences record containing linear, double-stranded DNA (186 bases) from Pontibacillus marinus = DSM 16465 strain BH030004. The record was created on November 12, 2014. It contains 1 feature. |
| AVBF00000000 | AVBF00000000.1 is a bacterial sequences record containing linear, double-stranded DNA (153 bases) from Pontibacillus yanchengensis strain Y32. The record was created on November 3, 2014. It contains 1 feature. |

**Strain ID Summary**

| Strain ID | Summary |
| --- | --- |
| NRRL B-59408T | Collection Code: NRRL  Collection Name: Agricultural Research Service Culture Collection  Institution: NRRL (Agricultural Research Service Culture Collection) - The United States  Strain ID: NRRL B-59408T |
| CGMCC 1 | Collection Code: CGMCC  Collection Name: China General Microbiological Culture Collection Center, Chinese Academy of Sciences  Institution: CGMCC (China General Microbiological Culture Collection Center, Chinese Academy of Sciences) - China  Strain ID: CGMCC 1 |

**Reference Search Summary**

| Name | Occurence |
| --- | --- |
